# Ractopamine at the Center of Decades-Long Scientific and Legal Disputes: A Lesson on Benefits, Safety Issues, and Conflicts

**DOI:** 10.3390/biom12101342

**Published:** 2022-09-21

**Authors:** Kumail Abbas, Aqeel Raza, Ross D. Vasquez, Marri Jmelou M. Roldan, Nemi Malhotra, Jong-Chin Huang, Olivia E. M. Buenafe, Kelvin H. -C. Chen, Shih-Shin Liang, Chung-Der Hsiao

**Affiliations:** 1 Department of Bioscience Technology, Chung Yuan Christian University, Taoyuan 320314, Taiwan; 2 Department of Veterinary Medicine, Faculty of Veterinary Science, Chulalongkorn University, Bangkok 10330, Thailand; 3 Research Center for the Natural and Applied Sciences, University of Santo Tomas, Manila 1015, Philippines; 4 The Graduate School, University of Santo Tomas, Manila 1015, Philippines; 5 Department of Pharmacy, Faculty of Pharmacy, University of Santo Tomas, Espana Blvd., Manila 1015, Philippines; 6 Department of Applied Chemistry, National Pingtung University, Pingtung 900391, Taiwan; 7 Department of Chemistry, Ateneo de Manila University, Katipunan Ave., Loyola Heights, Quezon City 1108, Philippines; 8 Department of Biotechnology, College of Life Science, Kaohsiung Medical University, Kaohsiung 80708, Taiwan; 9 Institute of Biomedical Science, College of Science, National Sun Yat-sen University, Kaohsiung 80424, Taiwan; 10 Department of Medical Research, Kaohsiung Medical University Hospital, Kaohsiung 80708, Taiwan; 11 Center for Nanotechnology, Chung Yuan Christian University, Taoyuan 320314, Taiwan; 12 Research Center for Aquatic Toxicology and Pharmacology, Chung Yuan Christian University, Taoyuan 320314, Taiwan

**Keywords:** ractopamine, feed additive, toxicity, aquatic animals

## Abstract

Ractopamine (RAC) is a synthetic phenethanolamine, β–adrenergic agonist used as a feed additive to develop leanness and increase feed conversion efficiency in different farm animals. While RAC has been authorized as a feed additive for pigs and cattle in a limited number of countries, a great majority of jurisdictions, including the European Union (EU), China, Russia, and Taiwan, have banned its use on safety grounds. RAC has been under long scientific and political discussion as a controversial antibiotic as a feed additive. Here, we will present significant information on RAC regarding its application, detection methods, conflicts, and legal divisions that play a major role in controversial deadlock and why this issue warrants the attention of scientists, agriculturists, environmentalists, and health advocates. In this review, we highlight the potential toxicities of RAC on aquatic animals to emphasize scientific evidence and reports on the potentially harmful effects of RAC on the aquatic environment and human health.

## 1. Introduction

Feed additives are nonnutritive products added to the basic feed mix to enhance productive function and growth, preserve feeds, increase the efficiency of feed utilization, or benefit metabolism and animal health [1,2]. Numerous studies and individual experiences gained by livestock owners have shown that the comprehensive feeding of animals, especially for high-yielding cattle, is impossible without highly effective feed additives such as antibiotics [3,4]. The beneficial effect of antibiotics as a growth stimulant was discovered in the 1940s [1,2]. Their uses in aquaculture for disease control, prevention, and growth promoters have been practiced for a long time. However, the unrestricted and widespread use of prophylactic antibiotics in aquaculture has caused a series of developments harmful to human health and the environment [2]. The consumption of antibiotics as feed additives in large amounts increases the tendency of their residual products to settle in aquaculture ecosystems, accumulate in fish meat that compromises their immunity, and cause the emergence of antibiotic-resistant bacteria in aquatic environments [5,6].

RAC is a popular growth promoter extensively used as a feed additive for muscle leanness in cattle. RAC stimulates lipolysis, redirects nutrients from adipose tissue, and increases protein synthesis [7,8,9]. RAC and other β-adrenergic agonists were traditionally used to treat respiratory disorders and premature birth in human beings [10]. Before RAC was approved for use as an additive in some countries, it already passed an extensive approval process through the FDA to determine the no-observed-adverse-effect level (NOEL or NOAEL; 0.125 mg/kg/day) and the acceptable daily intake (ADI; 1.25 mg/kg/day) was completed in December of 1999 (FDA, 1999). Although RAC use as a growth stimulant was allowed in 2000, its use has always remained controversial [11]. RAC passed the approval processes from the FDA that are similarly applied for the approval of anabolic implants. This process was administered by FDA veterinarians, animal scientists, biologists, and toxicologists. This strict process leaves a convincing impression that RAC is safe for the health of the aquatic environment. 

**Figure 1:**
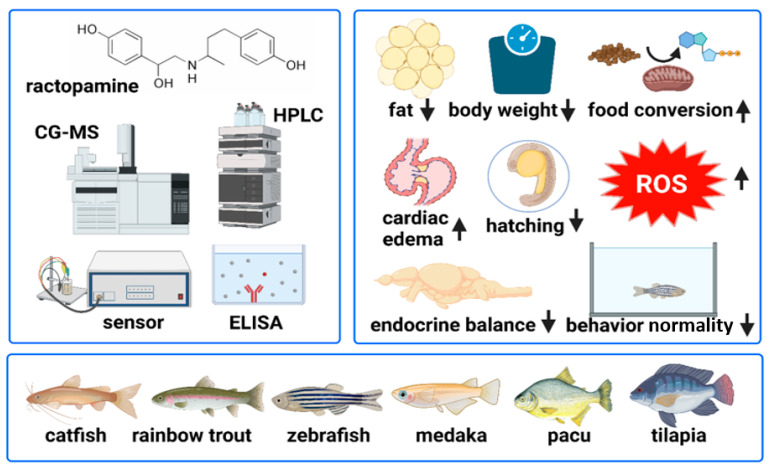
Summary of standard detection methods, aquatic animal models, and reported physiological alterations for ractopamine. The upper left panel summarizes the ractopamine chemical structure, standard detection instruments, and methods. The typical fish species used to study ractopamine’s beneficial and adverse effects are summarized in the bottom panel. The upper right panel summarizes the benefits and adverse effects of RAC administration in aquatic animals.

In this article, we have summarized the significant information on RAC’s potential benefits, toxicities as a feed additive, evidence on bioaccumulation, and the variation in methods used in quantifying its levels in Figure 1. This information is essential to understand why RAC’s use as a feed additive remains contentious and why this challenge will remain over the years. We highlight the prevailing scientific findings on the physiologic and toxicological effects of RAC on different fish species to understand the concerns of environmentalists advocating for the use of safe feed additives. 

## 2. Applications of RAC in Livestock and Poultry Sectors

RAC, a phenethanolamine β–adrenergic agonist, is used as a feed additive to develop leanness, enhance growth performance, and increase feed conversion efficiency in different farm animals, including poultry in some countries [12,13,14]. Presently, RAC is being used as a feed additive in livestock rations to decrease the fat contents in carcasses without changing their quality [15]. Consumers nowadays have a high demand for quality carcasses with minimal fat content, leanness, and tenderness, which can be achieved by adding RAC to the animal ration diet [16]. Numerous studies revealed improved feedlot performance and carcass composition when RAC is fed to poultry animals (Table 1). Previous studies suggest that adding RAC to an animal diet can lower feed intakes, but it improves the feed efficiency in swine when 10–20 ppm (parts per million) of RAC is added [16,17,18]. The introduction of RAC in rations also improves carcass protein and decreases the fat percentage in swine [18,19,20]. The addition of RAC in the finishing diet of pigs can regulate animal metabolism by optimal carcass muscle production and decrease the lipid concentration [21,22]. Adding RAC in the finishing ration of swine also enhances growth, feed conversion ratio, and carcass dressing percentage [23]. Several studies assessed the additive effects of RAC feeding in poultry animals. In the Parr et al. study (2011), a higher dose of trenbolone acetate combined with 17-β estradiol improved steer performance and hot carcass weight [24]. Bryant et al. (2010) compared the results of ractopamine and steroidal implants with varying trenbolone acetate and 17-β estradiol concentrations in finishing steers. They found out that the addition of one anabolic implant in holding days on feed constant resulted in an increase in the average daily gain (ADG) by 21%, 27% with two anabolic implants; and an additional 2% ADG increase with two anabolic implants and dietary administration of RAC for the last 28 days of feeding [25]. Aside from the positive effects on growth performance and carcass traits, RAC efficiently increased feed efficiency (gain-to-feed ratios) and carcass weight, with carcass weight increased by more than 100 pounds compared to control cattle [25]. In a meta-analysis of research data from more than 50 studies for both RAC and zilpaterol, dietary supplementation of RAC in cattle presented an increase in weight gain, hot carcass weight, longissimus muscle area, and efficiency of gain to feed [26]. 

**Table 1:** Summary of the physiological effects of ractopamine administration in livestock animals.

Feed Additives	Animals	Concentration	Physiological Effects	References
Ractopamine	Crossbred gilts and barrows	0 and 20 ppm	Improved feed efficiency, average daily gain (ADG), and decreased cooking loss of loin	[27]
Ractopamine	Pigs	20 mg/kg	Increased ADG, decreased feed conversion ratio (FCR), higher carcass lean proportion	[28]
Ractopamine	Pigs	0, 5, 10, and 20 mg/kg	Increased growth (*p* < 0.001), better efficiency (*p* < 0.001), and intensified muscular profile (*p* < 0.001)	[29]
Ractopamine	Dogs	1 mg/kg	Acute myocardial activity	[30]
Ractopamine and clenbuterol	Roundworm (*Caenorhabditis elegans*)	10 µg/L	Decreased brood size, alteration in locomotion behavior, reduced lifespan	[31]
Ractopamine	Cattle	200 to 350 gm/animal	Increased protein deposition and decreased lipogenesis, increased feed efficiency, increased ADG, and increased carcass weight	[26]
Ractopamine	Cattle	200 mg/animal/day for 28 to 42 days	Increased incidence of death (from 0.59 to 1.129/10,000 cattle)	[32]
Ractopamine	Pigs	0 mg/kg to 7.4 mg/kg	Increased ADG (18.8%), improved gain-to-feed efficiency (23.7%), increased carcass yield (0.7% units), and reduced backfat depths (6.3%) as compared to control (0 mg/kg)	[33]
Ractopamine + CON basal diet	Pigs	CON basal diet, CON + 1% ractopamine	Increased lean meat in the RAC group, fecal score, and growth performance was nonsignificant	[34]

The significant improvement in carcass quality due to RAC feeding is considered by meat producers as a looming economic benefit of hundreds of dollars, potentially overshadowing the perceived environmental problems of RAC uses. Despite the great potential of RAC as a growth stimulant, conflicting research results began to question the efficiency of RAC for the meat industry. Previous studies showed that growth performance declines as the duration of RAC feeding is prolonged, but an improvement in muscular growth continues with the increasing duration of RAC use [26,35,36,37,38]. Outputs from various studies also showed that including RAC in the finishing ration for swine improved ADG (*p* < 0.001) compared to untreated groups. Experiments from 1990 to 2005 showed an almost similar ADG improvement pattern when swine were given rations containing 5, 10, and 20 mg/kg RAC in the finishing diet [35,39,40,41,42]. However, two studies showed that pigs fed with RAC in the finishing ration had reduced ADG by about 0.9% [43] and 1.9%, respectively [44]. Feed intake of swine improved when the ration inclusion level was about 5 mg/kg for 34 days [35]. Carcass weight was increased by 2.3% when the finishing ration of pigs contained 5 mg/kg of RAC compared to 0 mg/kg RAC [35,42]. In similar studies, the addition of 10 mg/kg RAC in the dietary ration resulted in a decrease in hot carcass weight [17,45]. The addition of 20 mg/kg RAC can either reduce [27,46] or increase the carcass weight from 0.3% to 5.1% [42,47]. An increase from 4.4% to 10.7% in carcass weight was also reported when the pig was fed with 20 mg/kg RAC in the finishing ration before slaughter [35]. Other studies also reported 1.9% to 5.2% and 5.5% increases in carcass weight when the finishing ration included about 20 mg/kg RAC, respectively [39,41].

## 3. Biological Basis of the RAC Response in Animal Tissues

For more than 20 years, the potential for beta-adrenergic receptor (*β*AR) agonists to modify growth rate and body composition has been investigated. RAC is the first *β*AR ligand to be cleared for use in pigs in the United States. RAC is structurally similar to the natural catecholamines epinephrine and norepinephrine and binds with high affinity to βAR in pig adipose and muscle tissue. Primary attention has been given to understanding how βAR might mediate increased growth and protein accretion [48]. Catecholamines (CAs) are a group of organic compounds consisting of a hydroxyl group and an amine side chain. Most CAs are used by dopamine, norepinephrine, and epinephrine receptors in the nervous system [49,50]. CAs have received the attention of researchers because of their early recognized involvement in different neurological disorders [51]. The biological effect of CAs is mediated by two receptors, namely α- and β-adrenergic receptors, and sometimes they are collectively called adrenoreceptors. Alpha- and beta-adrenergic receptors have different locations in animal tissues and respond differently and often oppositely to catecholamines [52]. An adrenoreceptor stimulated by an α-agonist leads to intracellular effects mediated by adenylate cyclase inhibition. This stimulates smooth muscle contraction in blood vessels that supply peripheral organs such as skin and kidneys, smooth muscle relaxation in the gastrointestinal tract, and blood platelet aggregation. However, stimulating β-adrenergic receptors by β-agonists activates adenylate cyclase, leading to increased glycogenolysis and gluconeogenesis in the liver and skeletal muscle and increased lipolysis in adipose tissue [53]. Increased blood flow to the brain activates the catecholamine, making animals cope with stressful conditions such as reducing feed during the dry period, mixing animals in unfamiliar groups, transportation, and rough handling [54]. However, the measurement of CAs in urine and the brain cannot provide a clear picture of stress, fear, and temperament. Some scientists consider CAs a coping hormone as they provide energy to the brain and help reduce deficiency, leading to “energy deficiency syndrome” of the brain [55].

It is reported that RAC can steer the fat accumulation in the body of cattle and pigs via a prime metabolic channel through adipocyte accumulation and liberation by energizing β-adrenergic receptors because of similarities with catecholamine. As a result, minimal fat is deposited in the carcass. Animals fed with varying levels of RAC have improved carcass development and a lower percentage of adipose tissue compared to control animals [42,56]. Accumulating a lower percentage of fat in carcasses resulted as RAC directly affected adipocytes by increasing the rate of lipolysis and inhibiting the transformation of glucose to triglyceride [56,57,58,59]. Beta-adrenergic agents such as RAC orchestrate the cellular reaction via β-adrenergic receptors and trigger adenylate cyclase and protein kinase A, affecting the lipogenic activity by two mechanisms. The first one is protein kinase A, which directs the phosphorylation of existing proteins, decreasing their functional activity [60,61,62]. The second one is increased protein kinase A activity that reduces the rate of gene transcription and cell content of the main protein [63,64]. However, previous experiments revealed that swine fed with a RAC diet had decreased lipogenic enzyme activity in fat tissues [57,58,59]. The β-adrenergic agents affect the metabolism of adipose tissue through activation of β-receptors of protein kinase A. Increased protein kinase A activity results in increased lipolysis through activation of hormone-sensitive lipase [65]. Moreover, increased protein kinase A activity inhibits glucose conversion to triglycerides, which is considered increased serine phosphorylation. Lipoprotein lipase is an important enzyme that controls the triacylglycerol between the muscle and adipocyte tissue, improves lipid storage, and provides energy or muscle growth. Therefore, including RAC in diet changes lipid metabolism, inducing lipolysis rather than inhibiting lipogenesis in the animal. In summary, strong science supports the use of RAC as feed additives. RAC is an energy repartitioning agent that diverts nutrients by increasing protein synthesis ratio and decreasing protein degradation, promoting muscle growth by inducing muscle hypertrophy, reducing fat deposition, improving feed conversion, and increasing average daily weight gain that improves carcass yield and meat quality. 

## 4. Potential Benefits of RAC Feeding on Fishes 

The fast development in aquaculture systems is managed by different factors, including the growing consumption of formulated aqua-feeds and a massive improvement in the culture systems [66,67]. Phenethanolamines (PEOHs) act as a nutrient dissemination agent in intermediary metabolism by transferring nutrients from adipocytes to muscle protein unification [56,68,69]. The rationing of PEOH in fishes is not a common practice as fishes have lower energy demand and thus respond to diets with a higher protein–energy ratio than birds and mammals [70]. Previous data showed that RAC supplementation in channel catfish (*Ictalurus punctatus*) promoted weight gain and reduced fat deposition [71]. RAC feeding at 20 mg/kg or lower resulted in a 17% increase in weight gain and a 24% reduction in muscle fat in catfish [72]. In combination with dietary protein supplements in catfish, RAC yielded higher weight gain than when RAC was only combined with restricted protein supplementation [73]. In varying concentrations, RAC has been found to increase the feed efficiency in rainbow trout (*Oncorhynchus mykiss*) (walbaum) while maintaining normal hepatosomatic and viscerosomatic indices throughout the feeding weeks [74]. However, this RAC efficiency was not observed in small rainbow trout and channel catfish fed with RAC-supplemented dietary protein [75]. The same authors also reported that RAC is ineffective in bigger fish as rearing temperature plays a major role in growth and development. In another study, the combined effect of RAC at 0 and 10 mg/kg and l-carnitine at three levels (0, 1, and 2 g/kg) showed that 1 g/kg l-carnitine and 10 mg/kg RAC improved growth performance, feed efficiency, and protein efficiency ratio in juvenile rainbow trout. The combined use of l-carnitine and RAC in trout diets increased body protein, reduced body fat, and altered the fatty acid profile of muscle tissue [76]. Increasing levels of RAC in the diet of juvenile pacu (*Piaractus mesopotamicus*) for 60 days did not improve body growth or composition but only altered the hematological and biochemical parameters [77]. Pacu (*Piaractus mesopotamicus*) fed with 33.75 ppm of RAC in the finishing phase developed less fat content in its meat and improved antioxidant status inside the freezer [78]. On the other hand, the inclusion of RAC in the feed diet of Nile tilapia (*Oreochromis niloticus*) and tambaqui (*Colossoma macropomum*) for 31 days at varying inclusion rates did not alter the body composition, metabolism rate, and fat content [79,80]. 

## 5. RAC Level Detection in Wastewater Systems

Veterinary drugs in water bodies have become an alarming issue for nontargeted species and human populations [81,82,83]. The excessive use of veterinary drugs and steroidal hormones in animal feeding systems is viewed as one factor that negatively affects the health of aquatic species. RAC could be introduced into aquatic systems from water overflow areas rich in toxic waste from ruminants and is the most commonly detected drug (130–500 ng/L) in pond wastewater at pig farms [84]. RAC was also seen at 50 ng/L in groundwater due to run-offs from ruminants’ waste control facilities [84]. In China, another β-agonist, clenbuterol, used in treating respiratory illness and premature birth in humans, was identified at a concentration of 11 ng/L in abattoir wastewater [85]. Hospital sewage is one of the aquatic system’s major sources of β-agonists. In Taiwan rivers, the presence of four β-agonists from hospital discharge was reported, and RAC, at 70% prevalence, is the most common antibiotic present in the collected samples [86]. Due to the frequent detection of β-agonists in environmental water samples, researchers from various fields have initiated investigations to determine toxic compounds such as RAC. The occurrences of β-agonists in our environment are at a lower concentration under parts per million or parts per billion. Their intractable chemical behaviors made their detection very difficult, and their detection always poses analytical challenges. Due to the diversity of their physio-chemical features and continuing occurrence of β-agonists in our water, devastating consequences on both human and aquatic lives are expected in the near future. 

## 6. RAC Poses Physiological and Toxicological Effects on Fishes 

Data on the potential toxicity of RAC are limited. However, most research on aquatic toxicology evaluates the acute and chronic effects of RAC in nontargeted aquatic animals such as fishes such as medaka and zebrafish (Table 2). One study in medaka fish (*Oryzias latipes*) showed that chronic exposure to RAC did not affect the growth pattern, hatching time, and body parameters. However, it disturbed the endocrine system, altered transcription of genes related to the HPG axis pathways, and affected the antioxidative ability of female fishes [87].

**Table 2:** Summary of the physiological and toxicological effects of RAC administration in fishes.

Feed Additives	Animals	Concentration	Physiological or Toxicological Effects/Findings	References
Potential beneficial effects				
Ractopamine	Channel catfish (*Ictalurus punctatus*)	0, 20, and 100 mg/kg	Increased weight gain and reduced fat deposition	[71]
Ractopamine and dietary proteins	Channel catfish (*Ictalurus punctatus*)	RAC = 0 and 20 mg/kg Protein = 240 and 360 g/kg	Increased weight gain and less fat deposition are more functional when surplus protein is ingested	[73]
Ractopamine	Rainbow trout (*Oncorhynchus mykiss*) (walbaum)	0, 5, 10, 20, and 40 ppm	Higher feed efficiency in average treatment weeks No effect of RAC on growth, feed intake, and efficiency after 8–12 weeks of trial. No effect of RAC on hepatosomatic index (HIS)	[74]
Ractopamine and dietary proteins	Rainbow trout (*Oncorhynchus mykiss*) (walbaum)	RAC = 0 and 10 ppm CP = 25%, 35%, and 45%	Protein level more significantly affects growth, carcass composition, and pigmentation than ractopamine	[75]
Ractopamine and l-carnitine	Rainbow trout (*Oncorhynchus mykiss*)	RAC = 0 and 10 mg/kg l-carnitine = 0, 1, and 2 g/kg^−1^	1 g/kg l-carnitine and 10 mg/kg RAC enhanced the specific growth rate, feed efficiency, FCR, protein efficiency of fish, increased serum albumin level, total protein, and globulin	[76]
Ractopamine	Juvenile pacu (*Piaractus mesopotamicus*)	RAC = 0, 10, 20, and 40 mg	Feeding RAC for 60 days did not improve growth and body composition at any tested concentration but altered hematology and biochemical parameters	[77]
Ractopamine	Pacu (*Piaractus mesopotamicus*)	RAC = 11.25, 22.50, 33.75, and 45 ppm	RAC at 11.25 ppm reduced the fat content in fillets of pacu but improved the peroxide formation in samples kept in the freezer for 60 days. At 33.75 ppm, RAC was potent in preventing oxidation during storage in the refrigerator	[78]
Ractopamine	Nile tilapia (*Oreochromis niloticus*)	RAC = 0, 4, 8, 12, and 16 mg/kg	RAC showed a limited effect in changing body composition, lowering fat contents, and no changes in growth parameters when fed for 31 days	[80]
Ractopamine	Tambaqui (*Colossoma macropomum*)	RAC = 0, 2.5, 5, 10, and 20 mg/kg	RAC showed a limited effect on changing metabolism and reducing fat content. However, using a 20 mg/kg RAC dose for 30 days induced a slight decrease in visceral fat	[79]
Potential adverse effects				
Ractopamine	Japanese medaka (*Oryzias latipes*)	5, 25, 125, and 625 ppb	Disruption of the endocrine system and antioxidative/detoxification genes were affected	[87]
Ractopamine	Zebrafish (*Danio rerio*)	0.1, 0.2, 0.85, 8.5, and 85 ppb	Behavioral alteration and oxidative status imbalance	[88]
Ractopamine	Zebrafish (*Danio rerio*)	0.1, 0.2, 0.85, 8.5, and 85 ppb	Increased cardiac rate, induced exploratory behavior, no influence on hatching and survival rate	[89]
Ractopamine	Zebrafish (*Danio rerio*)	250, 350, and 450 ppm for 21 days	Behavioral alteration compromised the reproduction ability of adult zebrafish. Heart edema, granular formation, delayed hatching, and abnormalities in embryos	[90]
Ractopamine	Zebrafish (*Danio rerio*)	0.1, 1, 2, 4, and 8 ppm for 24 h	Induced hyperactivity in zebrafish larval locomotory behavior, increased cardiac, blood flow, and oxygen consumption rates, beta- blocker (propranolol), after co-incubating with RAC, tends to normalize the induced hyperactivity at 8ppm, lowered the cardiac rate as a “rescue agent”	[91]

The utility of the zebrafish model for evaluating RAC toxicity is very well recognized because the manifestations of toxicity are widely demonstrated in this model [92]. For instance, RAC exposure of zebrafish at different concentrations for seven days exhibited altered behavior and imbalanced oxidative status [88]. Zebrafish larvae acutely exposed to RAC displayed altered heart rate and locomotory and exploratory behavior but maintained their survival rate [89]. In addition, adult male zebrafish exposed to RAC for 21 days revealed poor reproduction and breeding capability and altered behavior [90]. In the same study, the mating of RAC-fed male adult zebrafish with non-RAC-fed female adult zebrafish resulted in delayed hatching (72 hpf) and a significant number of abnormal embryos with stunted development, edema of the heart, granule formation, degenerated yolk, and yolk deformities.

Our previous study reported that RAC triggered the locomotory behavior of zebrafish larvae and induced hyperactivity in terms of the totality of distance covered, rotational movements, and burst count [91]. The same paper showed that RAC at 8 ppm affected cardiac physiology and output physiology, increased the oxygen consumption rate, and amplified blood flow velocity and heart rate. In addition, in silico molecular docking revealed that RAC has more binding affinity with ten β-adrenergic receptor subtypes in zebrafish than β-blocker propranolol which was used as a “rescue agent” in reversing the behavioral and physiological changes that were induced in zebrafish larvae before ractopamine feeding. Although these findings are in silico, they potentially suggest the harmful effects of RAC on the cardiovascular, respiratory, and locomotory physiology of aquatic animals other than zebrafish. To summarize, it is evident that different fish species’ potential physiological and toxicological responses after acute and chronic exposures to RAC are well documented. Multiple endpoints such as locomotor activity, oxygen consumption, cardiovascular performance, reproductive performance, and growth performance are used as parameters to assess the effects of RAC on various fish species in consideration of age, weight, size, and maturity. These data provide solid in vivo evidence to support that RAC plays a vital role in modulating cardiovascular, locomotory, and respiratory physiology in fishes. 

## 7. RAC Levels Detected in Poultry Animals and Products

Different scientific procedures have been used to determine and quantify RAC and other β-agonists in human body fluids, animal tissues, and products (Table 3). Amendola et al. utilized a GC-ion trap to select precursor and product ions in monitoring the clenbuterol spike in human urine [93]. Nanoparticles were also utilized to extract and detect RAC and salbutamol by an electrochemical process. Rajkumar et al. used glassy carbon electrode-modified poly taurine/zirconia nanoparticles (ZrO_2_) to identify RAC and salbutamol in swine muscle and human urinary samples [94]. With an additional derivatization procedure, HPLC-UV was a popular method to detect RAC in porcine muscle and urine samples [95,96,97]. For fast screening and monitoring of β-agonists, enzyme-linked immunosorbent assay (ELISA) is a convenient technique to quantify RAC [98,99]. An online stacking capillary electrophoresis method was also developed to quantify RAC due to a positive amine functional group in its structure [100]. 

**Table 3:** Summary of the methods used to measure ractopamine content in animals.

Specimen	Instrumentation	No. of β-agonists/Internal Standards	Linear Range	LOD/LOQ	References
Urine	GC–MS^3^ (electron impact–ion trap)	Clenbuterol/ methyltestosterone (IS)	0.5–5 ppb	0.2 ppb	[93]
Pig muscle and human urine	Electrochemical detection	Ractopamine and salbutamol/--	1–28 μM (ractopamine) 5–220 μM (salbutamol)	--	[94]
Pig muscle and urine	HPLC-UV	Ractopamine/ephedrine hydrochloride	0.01–2 ppm	LOD: 0.003 ppm LOQ: 0.01 ppm	[97]
Pig samples	HPLC-UV	Ractopamine, clenbuterol, and salbutamol	0.5–50 ppb 0.5–50 ppb 0.2–20 ppb	LOD: 0.1, 0.1, and 0.05 ppb	[96]
Pork	HPLC-UV	Derivatized ractopamine	0.15–100 μg/g	LOD: 0.078 μg/g	[95]
Pork	ELISA	Salbutamol and ractopamine/--	0–1.0 ppb	LOD: 0.5 ppb	[98]
Swine meat/animal feed	ELISA	Salbutamol/--	0.05–1.0 ppb	LOD: 0.3–1.5 ppb LOQ: 0.6–3.0 ppb	[99]
Porcine meat	MEKC	Ractopamine	10–300 ng/g	LOD: 5 ng/g	[100]
Animal feeds	HPLC-MS	Ractopamine, clenbuterol, and salbutamol	0.5–500 mg/kg	LOD: 0.01 mg/kg LOQ: 0.05 mg/kg	[101]
Goat, various tissues	UPLC–MS/MS	Salbutamol/SAL-d_3_	0.5–100 ppb	LOD: 0.2 ppb LOQ: 0.5 ppb	[102]
Animal feeds	UPLC–MS/MS	Ractopamine, salbutamol, terbutaline, fenoterol, metaproterenol, clenbuterol, formoterol, tulobuterol, phenylethanolamine A/d_3_-salbutamol, d_6_-ractopamine, d_6_-clenbuterol	5–100 ppb	LOD: 0.01–0.05 ppb LOQ: 0.03–0.20 ppb	[95]
Goat, various tissues	UPLC-Q-Orbitrap	RAC/[d_6_]-RAC	0.5–500 ppb	LOD: 0.15 ppb LOQ: 0.5 ppb	[103]
Pork, beef, mutton, and chicken	UPLC-Q-Orbitrap	Salbutamol, cimaterol, bromchlorbuterol, ractopamine, isoxsuprine, mapenterol, terbutaline, cimbuterol, clenbuterol, brombuterol, mabuterol, clorprenaline/clenbuterol-d_9_, salbutamol-d_3_	0.01–50 ppb	LOD: 0.0033–0.01 ppb LOQ: 0.01–0.03 ppb	[104]

Abbreviation: LOD, limit of detection; LOQ, limit of quantitation; GC–MS, gas chromatography–mass spectrometry; HPLC-UV, high-performance liquid chromatography—ultraviolet; ELISA, enzyme-linked immunosorbent assay; MEKC, micellar electrokinetic chromatography; UPLC, ultra-performance liquid chromatography.

Nowadays, RAC levels can be estimated by high-performance liquid chromatography (HPLC) coupled with MS detection or HPLC coupled with tandem MS (MS/MS) [101]. Ultra-pressure liquid-phase chromatography (UPLC) coupled with triple-quadrupole MS was utilized to detect RAC within 5 min [95,102]. In addition, the mass analyzer, invented in 2002, provided excellent mass resolution and higher sensitivity qualities for detecting RAC levels [103,104]. 

The absence of a global standard method for RAC detection is one of the major issues for accepting RAC as a feed additive. This issue is due to several factors, and one major factor is economics. Some meat industries can cover the costs of mass spectrometry offered at reduced prices per unit by some testing laboratories. However, the high costs of the imported ELISA kits for the confirmatory test are still a burden [105]. At this point, the approval of confirmatory monitoring of animal products and techniques for RAC detection could be a strategic decision point. We emphasize the benefits, potential, and limitations of different state-of-the-art analytical procedures and their performance characteristics for RAC detection. The most obvious observation is that (reversed-phase) liquid chromatography combined with tandem mass spectrometric detection—either triple-quadrupole or ion-trap multi-stage—is the preferred technique in most cases, and member countries could adopt this to standardize their MRL or limit of tolerance. 

## 8. RAC Regulations and Feed Fights 

Different regulatory organizations such as the United States Food and Drug Administration (US FDA) and the European Medicinal Agency (EMA) and independent organizations such as the Joint World Health Organization/Food and Agricultural Organization Expert Committee on Feed Additives (JECFA) have determined tolerances and maximum residue limits (MRLs) of antimicrobials and growth stimulants such as RAC, respectively, for muscle, liver, kidney, and fat contents [106]. The MRLs derived by JECFA are recommended to the Codex Alimentarius Commission (Codex), which determines whether to establish international standards for residues of veterinary drugs in terms of MRLs. The US FDA uses the term tolerance, while other countries and organizations use MRLs. Other developed countries that are not part of the EU develop their own MRLs, while most developing countries adopt EU or Codex MRLs [107]. 

At present, RAC is the most controversial food additive in the world. It is well accepted that RAC has been authorized as a feed additive in many countries for the pig and cattle industry. The acceptability of RAC as a feed additive was based on the joint FAO/WHO Expert Committee on Food Additives and Scientific Evidence recommendations. However, it did not achieve a satisfactory judgment result between the European Union (EU) and the United States by the World Trade Organization (WTO) about the residual dosage of RAC, which could cause personal injury. Most jurisdictions, including the European Union (EU), China, Taiwan, Korea, and Russia, have disallowed its use for safety reasons [108]. This legal division reflects the long-standing disagreement between countries supporting the establishment of maximum residue levels and those who oppose it within the Codex. The Codex, an intergovernmental food standard-setting body with more than 180 members, ratified an MRL for RAC at 10 parts per billion (ppb) in pork and beef muscle meat [109]. Specifically, the Codex has recognized RAC maximum residue limits of 90, 40, 10, and 10 μg/kg for kidney, liver, fat, and meat, respectively [50]. The levels agreed upon were significantly higher than what many countries wanted. Countries such as the United States and Canada favored this decision with higher tolerances. However, the policy made it significantly difficult for China, Taiwan, and the European Union, which have a zero-tolerance policy. Meanwhile, the FDA set the MRL of ractopamine at 50 ppb for pork and 30 ppb for beef, significantly higher than the levels set by the Codex [110]. Currently, there is no evidence in the scientific community to prove that RAC is safe.

In 1998, the European Third-Country Directive included RAC in the list of banned substances. This ban is one of the most fiercely contested issues in the world. [111]. This directive prohibits using synthetic anabolic agents and importing implant-treated animals and meat parts to which implants were administered [112]. This ban was implemented despite conclusions published in several reports by a Scientific Working Group of 22 notable European scientists formed by the Commission of the European Communities that disproved any human health consequences of using anabolic growth stimulants in livestock production. The continuing international debate over RAC prohibitions, limits, and maximum residue level standards has deepened over the years, and a trade war may be possible in the near future [113]. 

The use of RAC also faces challenges posed by animal welfare organizations. Trade organizations for the meat industry contend the drug is proven scientifically to promote growth safely. In contrast, animal welfare groups argue that the drug harms both animals and humans. Animal welfare organizations contend that the FDA approved the higher MRL of RAC in livestock feed as a regulatory opportunity that favors the meat industry, not in the interest of animal health.

The international trade issue of RAC meat appeared as early as 2007 [107]. Undeniably, various barriers to trade, such as banning the use of growth compounds, unfavorably affect export markets and generate disputes among other countries. International trade continues to dominate how much RAC is used in livestock production. For example, Taiwan requires the United States to purchase electronic products, and the United States requires Taiwan to open the import of RAC meat in exchange. Issues on RAC importation could greatly affect the international relationship between these countries. It poses new problems and local disputes due to citizens’ protests and local governments’ resistance to the central government’s import decision on American pork containing RAC. Therefore, the relationships among central governments exist, which allow import, but local self-government formulates rules (or regulations) to reject central government order, and local government cooperates with citizens protesting import. In conclusion, this issue will remain problematic unless the legislative basis between the central government and local self-government is reconciled. The risk is adequately communicated to the citizen to minimize the opposing opinion about meat import, and inspection results are published and disclosed truthfully to citizens. 

Another reason for deepening the opposition is that the exporting countries are unwilling to take responsibility for marking RAC upon meat products, labeling warnings, or building up the compensation mechanism for personal injury, leaving customers who may become victims with no way to claim.

At present, the RAC controversy persists, and countries worldwide are divided on whether to allow or ban the use of RAC in meat production. In the United States, RAC is approved for swine, turkeys, and cattle [11,114]. RAC is also approved for Brazil, Canada, South Korea, and Mexico, but RAC has been banned in China, Taiwan, and the European Union. Despite an international CAC standard, there have been occasions in which exports from the United States to countries with zero-tolerance policies were rejected due to RAC levels under the global MRL [42]. One of the major arguments is the lack of consistency in how countries set their tissue RAC residue limits and which residue limits are applied to various tissues, specifically edible noncarcass [115]. The testing results from such countries can be contentious because they employ varying sample handling and testing methods that may impact the results. As presented in Table 2, diversities in technologies used to detect RAC reflect the inconsistency around how residue limits are established and which residue limits are applied to various tissues in different countries [115]. The CDC has not yet decided on the standards for the residues of RAC. It means that controversy still exists on the residual dosages of RAC meat, especially of the side effects of the cumulative doses of long-term intake of RAC meat in the human body. 

So, we learn from these conflicts considering that regulations governing RAC vary among countries and that the residues of RAC in animals can be exploited for risk assessment and monitoring illegal usage. It is highly suggested that exporting countries should guarantee food safety and use accurate methods to detect the negative result of RAC from cattle or swine before exporting to importing countries. For instance, exporting countries can guarantee food safety by utilizing Q-TOF/MS and LC-MS/MS assays of RAC. RAC glucuronide metabolites were identified in plasma and urine by quadrupole time-of-flight/mass spectrometry (Q-TOF/MS), and parent RAC residues were quantified in plasma, urine, and various tissues by LC-MS/MS (liquid) under the FDA recommended feed conditions, treatment days, and withdrawal days. This method detects oxolinic acid and RAC below the MRL in actual beef samples [116]. Therefore, this method is suggested for a surveillance system that reduces RAC usage and tracks its overuse for the future health of humans and animals. 

## 9. Conclusions 

Despite the controversy surrounding the use of beta-agonists as growth stimulants, the benefits to sustainability and animal production are apparent. However, the importance of production technologies such as RAC to meet the demands for quality meat of the growing global population cannot be overstated. RAC becomes a source of public concern and triggers endless transatlantic trade disputes. The threatening trade issues and accompanying abilities to detect extremely low concentrations of residues in tissues, variations employed by each country, and unscientific import policies could impact future RAC use. All these issues remain significant as RAC is still usually fed to livestock, and the potential toxicity of this substance is continuously being reported. Despite the looming economic benefits of RAC use, the potentially harmful and adverse effects on the environment and human health should not be overlooked and must be given attention. There is a great need for medical, scientific, pharmacological, and toxicological evidence from animal experiments to prove the causal relationship between RAC and its toxic effects on humans and animals. The present study emphasizes the urgent need to define a universal method of RAC monitoring at every stage of production, uniform or specific criteria on “level not limit,” toxicity tests, and tissue samples for analysis. It also emphasizes the need for a strict legislative and regulatory system in local and imported meat sectors where RAC is permitted and labeling compliance to protect human public health. 

## Data Availability

Any data not published within the article are available from the corresponding author on reasonable request.
